# Breaking barriers: evaluating access models for harm reduction vending machines

**DOI:** 10.1016/j.drugpo.2025.105079

**Published:** 2025-12-03

**Authors:** Ashleigh Herrera, Bradley Conner, Presleigh Beshirs, Ricky Bluthenthal

**Affiliations:** a California State University Bakersfield, Department of Social Work, 9001 Stockdale Highway, Bakersfield, CA 93311, United States; b Colorado State University, Department of Psychology, 1876 Campus Delivery, Fort Collins, CO 80523-1876, United States; c University of Southern California, Department of Population and Public Health Sciences, 1845 N. Soto Street, 3rd Floor, MC 9239, Los Angeles, CA 90032-3628, United States

**Keywords:** Harm reduction vending machine, Public health vending machine, Naloxone vending machine, Naloxone, Harm reduction supplies, Access models

## Abstract

The opioid epidemic continues to claim lives at alarming rates, disproportionately affecting marginalized communities, with structural barriers preventing equitable access to lifesaving interventions such as naloxone. Harm reduction vending machines (HRVMs) offer a promising solution to this issue, providing low-barrier access to naloxone and other harm reduction supplies. This study examines the impact of different HRVM access models on product utilization, specifically focusing on an outdoor HRVM, Project HOPE, located in Bakersfield, California. Initially, the machine required participant registration and imposed product limits; however, these restrictions were removed in August 2023 to improve access. An interrupted time series analysis of data from June to October 2023 reveal a significant increase in product utilization following the shift to unrestricted access, with increases in demand for naloxone kits, safer injection kits, wound care kits, and other basic needs supplies. The findings suggest that removing barriers to HRVM access, including registration requirements and product limits, can significantly improve the utilization of harm reduction supplies, potentially reducing opioid-related fatalities and promoting health equity. Further research is needed to evaluate the long-term impacts of unrestricted HRVM access models on overdose prevention and other health outcomes.

## Introduction

The opioid epidemic has prematurely ended the lives of over 1 million people in the United States (US; National Institute on Drug Abuse, 2023). While evidence-based approaches to overdose prevention such as improvements in the naloxone delivery cascade and medication for opioid use disorder have yielded an early promising decline in overdose deaths between April 2023, and April 2024, (CDC, 2025), drug-related overdose death vulnerability continues for people from minoritized groups (Kariisa et al., 2021). One explanation for this continued vulnerability among minoritized populations and unhoused or unstably housed people who use illicit drugs (PWUD) is the persistence of multiple structural barriers to obtaining and retaining naloxone (Goldshear et al., 2023). Recent research also has shown that Black and Latine people who use opioids were less likely to access naloxone training (Dayton et al., 2020) and possessed poorer naloxone cascade outcomes (e.g. receiving naloxone ever and within the past 6 months, obtaining naloxone refills after loss or use) compared to their White counterparts (Rowe et al., 2015).

To address these inequities, low-barrier, low-threshold harm reduction vending machines (HRVMs) - which dispense no-cost harm reduction, overdose prevention, and basic needs supplies - could be deployed to improve the naloxone delivery cascade among structurally vulnerable PWUDs. The first HRVM was implemented in Denmark in 1987, leading to widespread adoption internationally (Russell et al., 2023). Previous international studies established that HRVMs decreased syringe sharing (McDonald, 2009), HIV incidence (Arendt, 2023), and opioid-related fatalities following naloxone dispensation (Arendt, 2023; Allen et al., 2022), supporting the initial adoption in the US of HRVMs in 2017, and rapid proliferation in response to the US opioid epidemic.

To date, 537 HRVM sites operate in the US (excluding those located at universities) with at least one community-based HRVM operating in 43 states and Washington D.C. (see Supplemental Figure 1). However, the products available across HRVMs are heterogenous and subjected to US state and local laws related to syringe service program (SSP) operation and the criminalization of drug paraphernalia and drug checking equipment as well as organizational values and priorities. All 50 states in the US have laws allowing individuals to access and possess naloxone; therefore, naloxone is the only supply universally stocked in HRVMs. Other HRVMs offer naloxone and drug checking equipment (e.g. fentanyl and/or Xylazine testing equipment kits) (*n* = 107); naloxone and basic needs (e.g. hygiene, menstrual hygiene, wound care, safer sex kits, HIV/STI testing kits) (*n* = 25); naloxone, drug checking equipment, and basic needs (*n* = 122); or naloxone, drug checking equipment, basic needs, and safer substance use supplies (e.g. sterile syringes, safer injection supplies, safer smoking supplies, safer snorting supplies, or sharps disposal kits) (*n* = 75) (see Supplemental Table 1).

Despite the rapid expansion of HRVM programs across the US, HRVM program access models vary widely with limited evidentiary support for the programmatic decisions for this low-barrier harm reduction intervention. For instance, some HRVM programs require participants to register or complete a survey and create an identification (ID) number or receive a magnetic strip card to access supplies (*n* = 133). Similarly, HRVM programs nationwide do not implement a uniform approach to product limits. Some programs only permit participants to dispense a certain quantity of products per transaction or limit the frequency of use (e.g. once a day or once a week) (*n* = 144), and the rest provide unlimited product access (*n* = 392). The impacts of these programmatic decisions are largely unexamined, and research examining the effect of access models (e.g. restricted vs. unrestricted access) and product limits (limited vs. unlimited) on HRVM product dispensation is needed. To address these gaps, the present study examined the effects of restricted vs. unrestricted access models on product dispensation from an outdoor, community-based HRVM in Kern County, California. We hypothesized that product dispensation for all supplies would significantly increase during the unrestricted period.

## Methods

This naturalistic interrupted time-series design study had institutional review board approval.

### Setting

In June 2023, an outdoor, temperature-controlled HRVM (operated by Project HOPE, https://www.projecthopecoalition.org/resources) became operational in Bakersfield, California, in an area with low naloxone kit saturation (2.76 kits per 1000 residents) (Health Management Associates, 2023). The Project HOPE HRVM, available free to the community 24/7, is located outside a federally qualified health center (FQHC).

### Description of the machine

The Project HOPE HRVM was developed through a community-academic partnership between Clinica Sierra Vista (CSV), a FQHC, and California State University, Bakersfield (CSUB); a complete description of the implementation of the HRVM and the product kits can be found in our previous publication (Herrera, 2025). Based on the goal of improving equitable access to harm reduction supplies and previous findings on international best-practices for HRVM models (Russell et al., 2023), the partners elected to implement an outdoor HRVM accessible 24/7 to the public. The product kits in the Project HOPE HRVM included: nasal naloxone kits, safer sex kits, safer injection kits, wound care kits, sharps disposal kits, hygiene kits, and menstrual hygiene kits.

### The intervention

Between June and August 2023, only registered participants could access the Project HOPE HRVM with a personal ID number, and each user was subject to product kit limits. Participants could scan a QR code posted on the HRVM or register in-person with CSUB researchers. Participants were limited to the following dispensation schedule: once daily for safer-sex, safer-injection, wound care, naloxone, and menstrual hygiene kits; and once weekly for sharps disposal and hygiene kits.

The registration requirement for the HRVM program was removed in August 2023, due to limited participant uptake, barriers to independent registration, and the relocation of the Homeless Healthcare team that month from the adjacent clinic site. Additionally, limited organizational capacity made it difficult to sustain the registration process. As HRVMs are intended to be low-barrier, low-threshold interventions, registration and product limits were discontinued. Although no formal outreach campaign accompanied the change, increased community awareness and word-of-mouth may have contributed to greater use during the unrestricted access period. Product dispensation was tracked during the three months immediately following the implementation of unrestricted access (August through October 2023).

### Evaluation

The primary outcome was the number of each type of product kit dispensed by the HRVM from June to October 2023. Using descriptive statistics and an interrupted time series design, dispensation of harm reduction and health product kits was compared across restricted and unrestricted access periods. The HRVM launched on June 11, 2023, and shifted from restricted to unrestricted access on August 21, 2023, marking the interruption point. Interrupted time series analyses were run in SPSS Version 29.0 (IBM Corporation, 2025), with α = 0.05. All models were specified as autoregressive integrated moving average (ARIMA) models with parameters estimated by SPSS. Weekly counts of each product kit type and total dispensation were analyzed. A significant *t* for each series indicated whether removing restrictions significantly changed the dispensation pattern, with stationary R^2^ used to estimate effect size.

## Results

Over the study period, 995 product kits were dispensed from the HRVM. During the restricted period, dispensation totaled 162 kits (16 %), compared with 833 kits (84 %) during the unrestricted period (see Supplementary Table 1). While overall dispensation increased significantly post-restriction (*p* < .001), the relative distribution of product kit categories remained largely stable. Infection prevention kits comprised a larger proportion of total distributions, increasing from 29 % to 36 % (restricted: 4.7 kits/week; unrestricted: 50.3 kits/week), driven by growth in wound care kits (12 % to 22 %; 1.9 vs. 30.8 kits/week). Safer substance use kits remained proportionally stable at 34 % and 31 % (restricted: 5.5 kits/week; unrestricted: 42.5 kits/week), with safer injection kits representing 22 % in both periods (3.5 vs. 30.5 kits/week). Basic needs kits accounted for 30 % in both periods (4.9 vs. 42.0 kits/week). Overdose prevention kit dispensation declined proportionally from 7 % (restricted; 1.1 kits/week) to 3 % (unrestricted; 4 kits/week), reflecting relative distribution rather than a reduction in total volume dispensed.

Results of the interrupted time series analyses indicated significant increases in product kit dispensation following removal of restrictions (see Supplementary Figure 2). Safer sex product kits (0-order ARIMA; *t* = 4.47, *p* < 0.001, stationary R^2^ = 0.57), safer injection product kits (1st-order ARIMA; *t* = 12.04, *p* < 0.001, stationary R^2^ = 0.83), wound care product kits (0-order ARIMA; *t* = 8.75, *p* < 0.001, stationary R^2^ = 0.73), sharps disposal product kits (0-order ARIMA; *t* = 4.78, *p* < 0.001, stationary R^2^ = 0.38), menstrual hygiene product kits (0-order ARIMA; *t* = 5.77, *p* < 0.001, stationary R^2^ = 0.84), and naloxone product kits (1st-order ARIMA; *t* = 5.74, *p* < 0.001, stationary R^2^ = 0.69) all showed significant increases. Total product kit dispensation also increased significantly (0-order ARIMA; *t* = 7.59, *p* < 0.001, stationary R^2^ = 0.79).

## Discussion

Findings from the present study illustrate that the unrestricted access model increased HRVM product kit dispensation. While it is possible that several other factors could account for the increase in dispensation, such as more people learning about the machine and becoming more comfortable using it, lowering the threshold to access safe supplies likely accounts for a significant proportion of the increase in dispensation. Prior research indicates that unlimited, low threshold access to safe supplies is a reliable means to reducing health risks among PWUD (Tookes et al., 2024).

While naloxone kit dispensation increased during the unrestricted access model period, the demand for naloxone remained consistently low. A recent study of HRVM product dispensation in criminal justice settings also found low naloxone dispensation (Martin et al., 2025). However, limited awareness of overdose risk factors, inadequate knowledge of naloxone administration, and insufficient understanding of the HRVM program – along with concerns about potential criminal legal consequences associated with its use – may affect dispensation rates across HRVM programs. Furthermore, the low dispensation of naloxone during the unrestricted period of the present study as well as the unrestricted HRVMs studied by Martin and colleagues (2025) dispel concerns about the misuse/diversion of naloxone and support unrestricted access HRVM program models for naloxone.

Restrictions like requiring registration to utilize HRVMs also create barriers for structurally vulnerable PWUDs to accessing health and harm reduction supplies. In a recent study by Arendt (2023), participants were required to register and re-enroll in an Ohio HRVM program by calling the program phone number or in-person. The author acknowledged phone access as a potential structural barrier to program participation (Arendt, 2023). Similarly, in the restricted access period, program participants required a smart phone to scan the QR code and complete the registration independently if they were unable to register in-person with the research team. While one study found that 94 % of persons experiencing homelessness reported currently owning a cell phone, only 58 % currently owned a smart phone (Rhoades et al., 2017). Therefore, HRVM programs requiring participants to register should consider implementing both phone and QR code registration methods as well as other non-technology-based methods to improve accessibility.

Although unrestricted access models may enhance service reach and participant engagement, they also introduce important considerations related to equity, workforce and organizational capacity, program costs, and supply chain logistics that should be carefully evaluated when planning and implementing such approaches. For programs distributing sterile syringes through HRVMs, software settings should allow unrestricted access to ancillary supplies while limiting syringe and injection equipment access to registered SSP participants who enter a unique ID. A similar mixed-access model can be applied to promote unrestricted and unlimited access to overdose prevention supplies (e.g., naloxone, fentanyl test strips, and xylazine test strips), while maintaining restricted or limited access for other products (e.g., nicotine replacement therapy, safer smoking supplies, and emergency contraception).

Many HRVM programs in the US experience legal and structural barriers to providing access to harm reduction supplies. However, Western European countries, which adopted HRVMs almost four decades ago, model best practices for HRVM programs in the US to improve health equity and access among PWUDs. Continued advocacy for low-barrier, low threshold HRVMs across the US is imperative to reduce drug-related morbidity and mortality.

## Conclusion

Given the high level of stigma and shame experienced by PWUDs, the lowest barrier HRVM access models should be adopted to promote equitable access to harm reduction and basic needs supplies. Even though the outdoor Project HOPE HRVM was accessible to registered participants 24/7/365, dispensation remained low, especially for the naloxone kits. The discontinuation of the registration process and product limits resulted in a significant increase in dispensation of all harm reduction kits, including naloxone. Unrestricted access models for HRVMs stand to increase naloxone saturation, enhance health equity, and decrease fatal opioid ODs among structurally vulnerable PWUDs.

## Supplementary Material

1

2

3

Supplementary material associated with this article can be found, in the online version, at doi:10.1016/j.drugpo.2025.105079.

## Glossary

HRVM: Harm reduction vending machine
PWUD: Person(s) who use drug(s)
SSP: Syringe service program
US: United States
