# Supplementary material for: Breaking barriers: evaluating access models for harm reduction vending machines
Source: Int J Drug Policy. Author manuscript; Available in PMC 2026 Jul 4. (PMC13331765; doi:10.1016/j.drugpo.2025.105079)
Supplement: 1 [file NIHMS2186788-supplement-1.docx]

Supplementary Figure 2. Weekly Product Dispensation Before and After Implementation of Unrestricted Access to HRVM


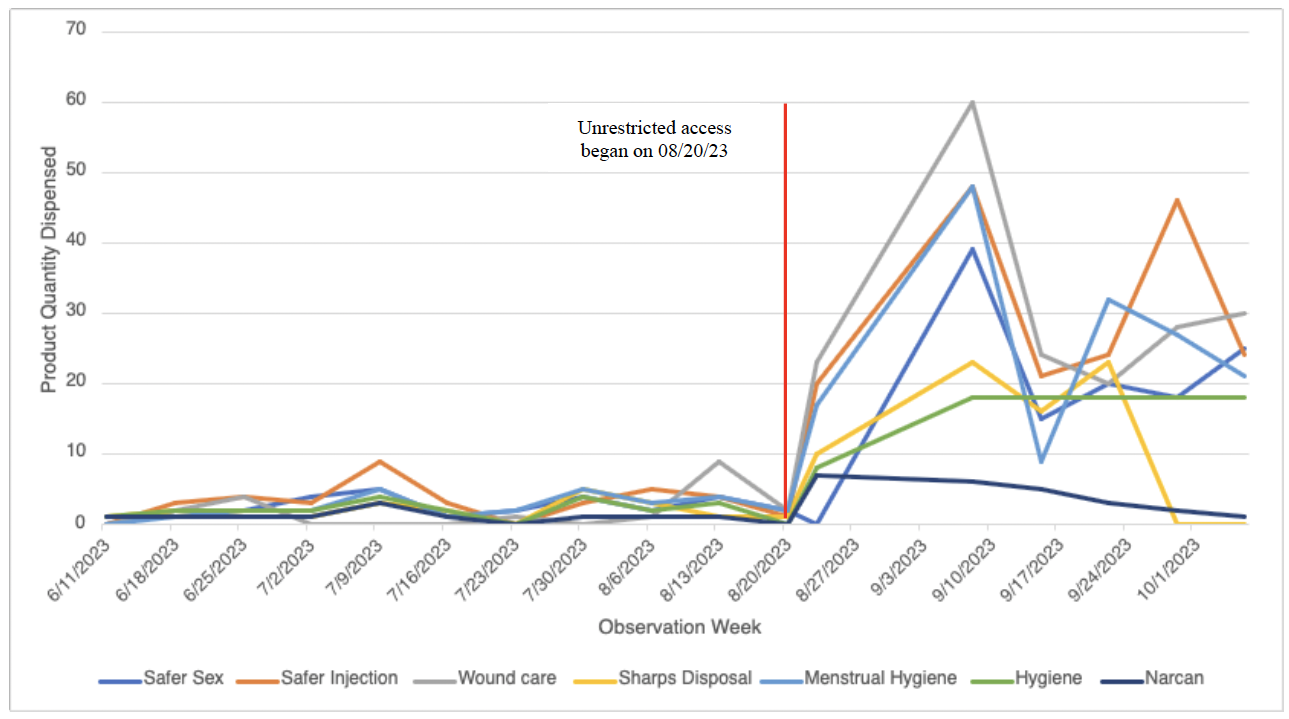


Caption. From June 11 to August 20, 2023, the HRVM operated under a restricted access model requiring participant registration and product limits. On August 20, 2023, the unrestricted access model was implemented. The line graph displays weekly dispensation patterns by product kit type across the project period.
