# Supplementary material for: Breaking barriers: evaluating access models for harm reduction vending machines
Source: Int J Drug Policy. Author manuscript; Available in PMC 2026 Jul 4. (PMC13331765; doi:10.1016/j.drugpo.2025.105079)
Supplement: 2 [file NIHMS2186788-supplement-2.docx]

Table 1.

| **Type of Item** | | **Restricted Access Model** | | **Unrestricted Access Model** | | **Total** |
| --- | --- | --- | --- | --- | --- | --- |
|  | | **n (%)** | **Weekly Average** | **n (%)** | **Weekly Average** |  |
| **Overdose Prevention Supplies** | Naloxone Kit | 11 (7%) | 1.1 | 24 (3%) | 4 | 35 (4%) |
| **Safer Substance Use Supplies** | Safer Injection Kit | 35 (22%) | 3.5 | 183 (22%) | 30.5 | 218 (22%) |
|  | Sharps Disposal Kit | 20 (12%) | 2 | 72 (9%) | 12 | 92 (9%) |
| **Infection Prevention Supplies** | Safer Sex Kit | 28 (17%) | 2.8 | 117 (14%) | 19.5 | 145 (15%) |
|  | Wound Care Kit | 19 (12%) | 1.9 | 185 (22%) | 30.8 | 204 (21%) |
| **Basic Needs Supplies** | Hygiene Kit | 22 (14%) | 2.2 | 98 (12%) | 16.3 | 120 (12%) |
|  | Menstrual Hygiene Kit | 27 (17%) | 2.7 | 154 (19%) | 25.7 | 181 (18%) |
| **Total** |  | 162 (100%) |  | 833 (100%) |  | 995 (100%) |

**Caption.** Number, percentage, and weekly average of product kits dispensed by type during the restricted and unrestricted access model periods, and total distribution across the project period. Each access model column presents the total number (n), the percentage (%) of all kits distributed within that period, and the average number dispensed per week. Weekly averages are not calculated for the total project period.
