# Supplementary figures and images for: Breaking barriers: evaluating access models for harm reduction vending machines
Source: Int J Drug Policy. Author manuscript; Available in PMC 2026 Jul 4. (PMC13331765; doi:10.1016/j.drugpo.2025.105079)

Supplementary Figure 1. HRVM locations and access models across the United States.

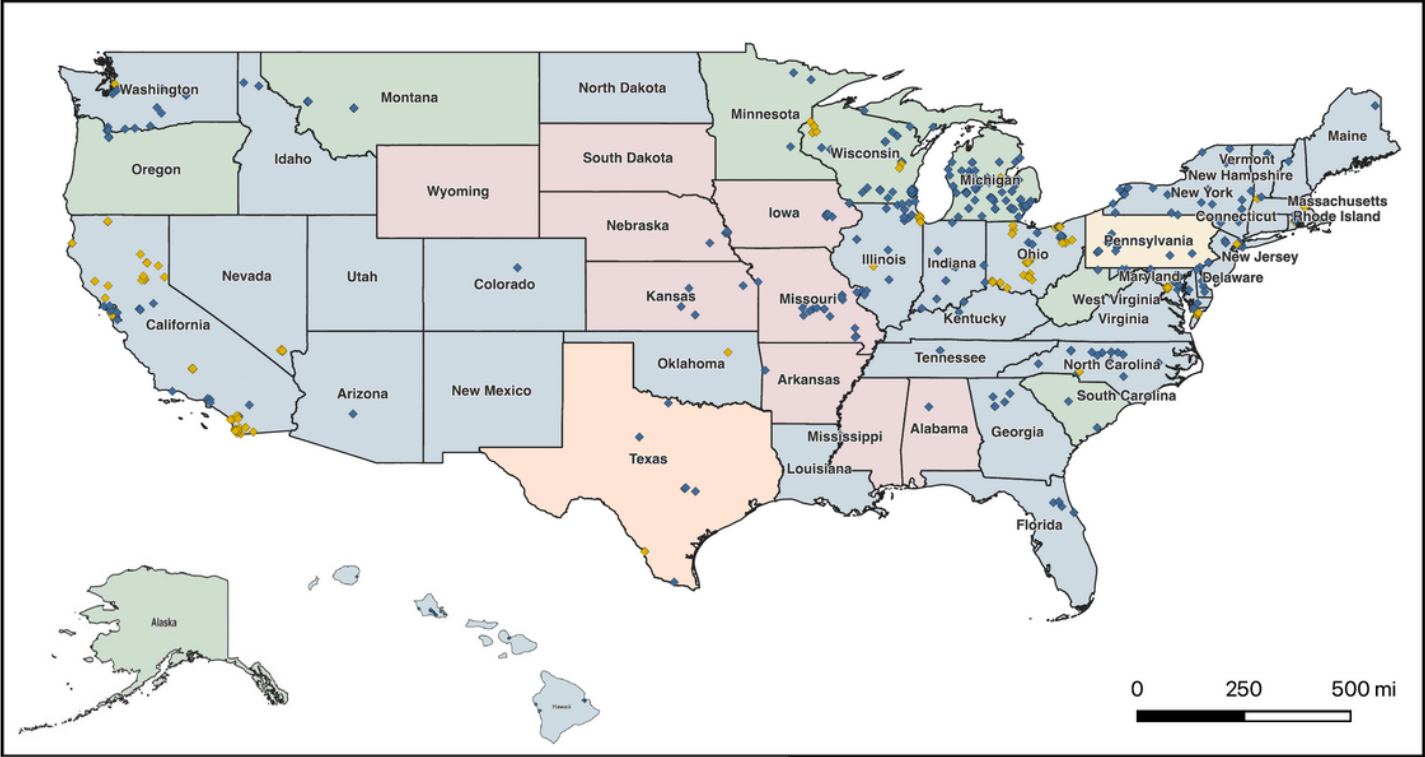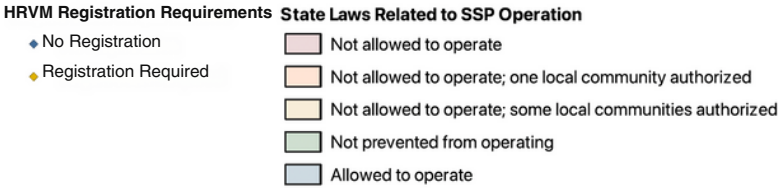

Supplement: 3 [file NIHMS2186788-supplement-3.pdf]
